# From quantum to continuum mechanics in the delamination of atomically-thin layers from substrates

**DOI:** 10.1038/s41467-020-15480-w

**Published:** 2020-04-03

**Authors:** Paul Hauseux, Thanh-Tung Nguyen, Alberto Ambrosetti, Katerine Saleme Ruiz, Stéphane P. A. Bordas, Alexandre Tkatchenko

**Affiliations:** 1 Institute of Computational Engineering Sciences, University of Luxembourg, L-4365 Luxembourg City, Luxembourg; 2 Dipartimento di Fisica e Astronomia, Università degli Studi di Padova, 35131 Padova, Italy; 3 Department of Physics and Materials Science, University of Luxembourg, L-1511 Luxembourg City, Luxembourg; 4 Cardiff University, School of Engineering, Cardiff, UK

**Keywords:** Method development, Mechanical engineering, Theory and computation, Nanoscale materials, Surfaces, interfaces and thin films

## Abstract

Anomalous proximity effects have been observed in adhesive systems ranging from proteins, bacteria, and gecko feet suspended over semiconductor surfaces to interfaces between graphene and different substrate materials. In the latter case, long-range forces are evidenced by measurements of non-vanishing stress that extends up to micrometer separations between graphene and the substrate. State-of-the-art models to describe adhesive properties are unable to explain these experimental observations, instead underestimating the measured stress distance range by 2–3 orders of magnitude. Here, we develop an analytical and numerical variational approach that combines continuum mechanics and elasticity with quantum many-body treatment of van der Waals dispersion interactions. A full relaxation of the coupled adsorbate/substrate geometry leads us to conclude that wavelike atomic deformation is largely responsible for the observed long-range proximity effect. The correct description of this seemingly general phenomenon for thin deformable membranes requires a direct coupling between quantum and continuum mechanics.

## Abstract (web summary)

The unexpectedly long-ranged interface stress observed in recent delamination experiments is yet to be clarified. Here, the authors develop an analytical approach to show the wavelike atomic deformation as the origin for the observed ultra long-range stress in delamination of graphene from various substrates.

## Introduction

Atomically thin membranes exhibit unique electronic and mechanical properties^1–4^ and promise transformative impact on fields as varied as stretchable nanoelectronics^5,6^, transparent electrodes^7^, flexible devices^8^, energy devices, sensors^9^, and nanocomposites^7^. The actual implementation of such devices depends on interfacial properties between atomically thin membranes and their substrates. In particular, the interaction distance range and energy between membranes and a given substrate are of fundamental importance to understand interfaces between layered materials^10^ and to develop effective manufacturing processes such as roll-to-roll transfer^11^, face-to-face transfer^12^, transfer printing^13^, or wet/dry transfer techniques^14^.

The different components in layered materials under normal conditions are often kept together through nonbonded van der Waals (vdW) interactions. Interaction distance ranges obtained through traditional models of vdW interactions are of the order of a few nanometers (more than 1 nm and definitely less than 10 nm). The delamination of a single graphene layer from a silicon substrate reported recently in ref. ^15^ (adhesive energy of 357 ± 16 mJ m^−2^) is commensurate with vdW interactions. However, the estimated force per unit surface area during delamination plateaus only at a distance of  ~1 μm. This result is in stark contradiction to all models of intermolecular interactions known to the authors, which predict an effective interaction range on the order of 2–10 nm, i.e. two to three orders of magnitude smaller than measured experimentally. Hence, it seems that even the fundamental nature and strength of the adhesive interactions between 2D materials and their substrates remain poorly understood.

For example, density-functional calculations with vdW corrections in ref. ^16^ obtain an interaction distance range of 1 nm. Molecular dynamics (MD) simulations of interfacial adhesion of a functionalized graphene surface and polyethylene in ref. ^17^ found an interaction range of 1.8 nm. Similarly, the dynamical interaction model proposed in ref. ^18^ with a sequence of morphological water transitions yields an interaction range of 3–4 nm.

We emphasize that beyond the graphene/silicon system, recent experimental literature contains a plethora of examples where ultra-long-range interatomic and intermolecular interactions have been observed. This includes delamination of graphene on copper foil in refs. ^19–21^, adhesion between graphene membranes and their substrates in ref. ^22^, adhesion of silicon over silicon^23^, molecules suspended over metal surfaces^24^, and bacteria over semiconductor protrusions^25^. In general, such long-range forces are thought to be largely responsible for the observed unique properties of nanocomposites, active colloids, and extended biological systems^26–28^.

Here, we aim to significantly improve our fundamental understanding of quantum-mechanical (QM) forces behind interfacial cohesion at the mesoscopic length scale. In particular, we study the delamination of one-dimensional (1D) nanostructures and two-dimensional (2D) graphene from substrates by unifying QM many-body treatment of microscopic vdW interactions with a continuum mechanics model for the elasticity and adhesive traction-separation law (TSL). We employ an efficient QM Hamiltonian applicable to thousands of atoms and carry out full variational relaxation of the coupled graphene/substrate system for separations up to 50 nm. We conclude that nanoscale cooperative effects involving many-body dispersion enhancement, and coherent wave-like mechanical deformations, are largely responsible for the observed ultra-long-range stress in delamination of graphene from various substrates. Remarkably, the observed emergent stress seems to be a rather general phenomenon for thin deformable membranes and nanostructures, stemming from quantum-mechanical many-body treatment of interatomic interactions beyond the standard pairwise models for the non-covalent interactions.

## Results

### Traction-separation law for graphene delamination

**Fig. 1:**
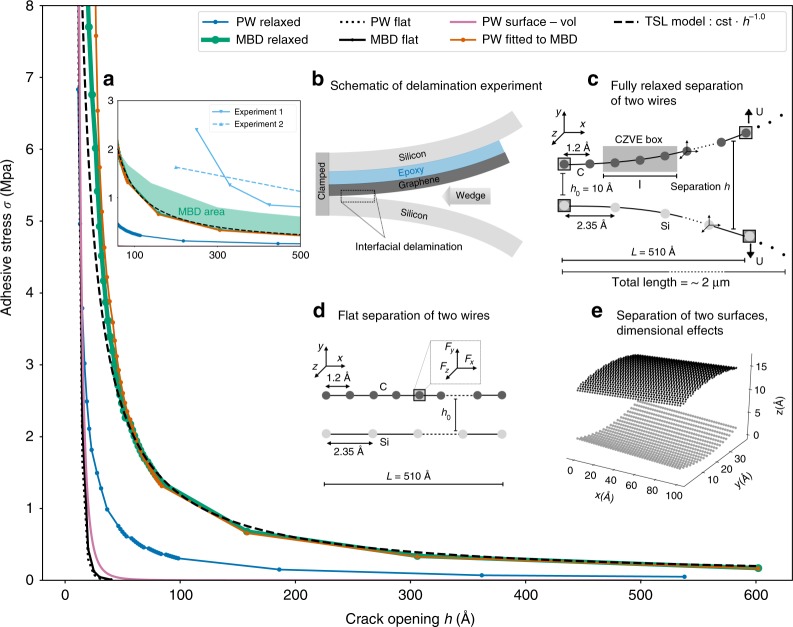
Adhesive stress between carbon and silicon obtained with different methods. Traction-separation law (TSL) calculated with numerical simulations with PW and MBD vdW interactions. The experimental setup for silicon/graphene/epoxy/silicon separation by using a test wedge, adapted from ref. ^15^ is schematically depicted in **b**. Three schematizations of the delamination model are proposed to study the adhesive interface: the first is based on flat separation (**d**), the second corresponds to the variationally optimized configuration (**c**), and the last scheme is an approximation of the relaxed geometry in 3D (**e**). Delamination along the *y* direction is accentuated in diagram (**e**) for clarity. In each scheme, both PW and MBD vdW approaches are adopted to describe nonbonded interactions. Atoms are separated by covalent bonds, which can be described by the pairwise harmonic potential. TSLs obtained numerically between graphene and silicon are plotted for the different models. Ultra-long-range interaction is found by variational optimization based on MBD interactions. Comparison with experimental data from ref. ^15^ is given in **a**. The MBD area indicates the sensitivity of MBD results with respect to the adopted damping parameters. The adhesive stress is computed exploiting the concept of cohesive zone volume element (CZVE), see details in “Methods” and Supplementary Note 3.

Compelling evidence and clear quantitative assessment of ultra-long-ranged proximity effects were recently reported upon experimental delamination of graphene from silicon, carried out in ref. ^15^. The schematic of the experimental setup is shown in Fig. 1b. In short, the insertion of a wedge opens a crack at the interface between silicon and graphene. The crack length is measured using infrared interferometry as a function of the wedge position. This information allows the calculation of the *J* integral^15^, a measure of the energy release rate, from which the adhesive stress *σ*(*h*) can be computed as a function of crack opening *h*.

As explained above, the main contribution to the adhesive energy in graphene-substrate systems is the vdW interaction between the two materials. Assuming that vdW interactions can be modeled in interatomic pairwise (PW) additive form, it is widely accepted that the distance range of such interactions is of the order of 1–4 nm^16–18^. This is in stark contrast with the experimental observations of nonvanishing *σ*(*h*) that extends at least to 500-nm distance range for graphene on silicon^15^.

To properly model complex adhesive phenomena, here we develop a comprehensive and hierarchical framework that bridges elastic and quantum-mechanical models for vdW interactions between the two materials with continuum descriptions of the TSL (see “Methods”). For the particular case of graphene on silicon, see Fig. 1 for an overview. Nearest-neighbor atom interactions are modeled by harmonic springs, which provide a faithful representation of chemical interactions in one-dimensional systems. Conversely, vdW interactions act between all atoms in the system (beyond a certain interatomic distance cutoff), either via the standard pairwise (two-body) Lennard–Jones (LJ) approximation, or according to a more accurate, explicitly quantum-mechanical many-body dispersion^29,30^ (MBD) model.

Our model can be generalized to a more sophisticated first-principles treatment of chemical bonds in a straightforward manner, and this remains an interesting avenue for future work. It is important to remark that the qualitative conclusions of this work are independent of the approximations we employ for the description of local elastic interactions, and it would be relatively straightforward to extend the current model to a more sophisticated treatment of local chemical interactions based on distances, angles, and torsions or embedded-atom potentials.

To describe the interfacial delamination experiments carried out in ref. ^15^, we first build a model involving two atomic wires. We consider a structure containing two interacting wires, namely a carbon chain (426 C atoms) and a silicon chain (218 Si atoms). Equilibrium interatomic separations in the initial state are ${r}_{0}^{{\rm{C}}-{\rm{C}}}=1.2$ Å and ${r}_{0}^{{\rm{Si-Si}}}=2.35$ Å. This leads to a structure of linear size *L* = 510 Å.

The simplest possible approach to calculate numerically the dependence of the adhesive stress on interwire separation consists of using a “flat-separation model” (see Fig. 1d). In this case, the geometry of each of the two atomic chains is constrained to remain flat as they are separated from each other, and the crack opening is simply defined as the distance between the two chains. The results show that the adhesive strength rapidly decays to zero when the crack opening exceeds ~35 Å, meaning that the interaction range is slightly larger than those obtained in the literature^16,18^, but remains three orders of magnitude smaller when compared with experimental observations. Importantly, the inclusion of quantum MBD interactions in a flat-separation model leads to a stronger adhesive stress for small crack openings (less than 15 Å), but yields only marginally larger adhesive range compared with PW calculations. We emphasize that despite apparently similar adhesive stress ranges, the mechanisms for the stress in MBD and PW methods are radically different even for flat chains. Due to the intrinsic locality of the PW energy and forces, the total vdW vertical force that acts on a carbon atom comes from the contribution of only a few silicon atoms directly underneath. All these contributions have a negative (attractive) sign. In contrast, within the MBD approach, both carbon and silicon atoms are involved in the total vdW vertical force that acts on a carbon atom. More importantly, an analysis of the different MBD contributions corresponding to the collective modes of plasma-like charge fluctuations in the system shows that they can have either positive or negative signs. These contributions also have a strongly nonlocal character. The balance between positive and negative terms ultimately determines the overall force and shows nontrivial dependence on the actual geometrical configuration. A detailed analysis of the flat model is provided in Supplementary Note 7 and Supplementary Figs. 4–7. For the flat-separation model, we also expect equivalent results as obtained in this work when using alternative analytic approaches to vdW and Casimir interactions^31–36^, given the qualitatively similar distance dependence of the dispersion forces in these models.

To achieve a more realistic description of the experiment, we also introduced a theoretical 3D approach for flat separation between a 2D membrane (graphene) and a bulk (silicon) substrate using the pairwise vdW potential, and compared the results with the flat-separation model involving two atomic wires. We obtain a slightly longer adhesive range with a 3D model; however, the adhesive stress still vanishes for a crack opening of ~45 Å. From this test, we conclude that a wire model, while being very approximate, can still reproduce at a semiquantitative level the dependence of the adhesive stress from the separation between layered materials.

Up to now, we have investigated the behavior of *σ*(*h*) for rigid geometries where all atomic positions match their equilibrium structure at infinite separation between materials. However, atomic relaxation due to the interaction between adhesive materials could be a decisive factor influencing *σ*(*h*)^37–40^. To investigate the effect of atomic relaxation, we developed a variational procedure based on energy and force minimization to characterize the fully relaxed equilibrium structure of two wires using PW and MBD methods (see “Methods”). The shape of the crack depends on the thickness and the speed of insertion of the wedge. In this paper, we model numerically the insertion of a thin wedge between two (initially parallel) chains at relative separation *h*_0_, by applying small vertical displacements (1.5% of the length of the chain) to the right-hand side edge atoms (see Fig. 1c), while maintaining left-hand edge atoms fixed. To compute the TSL with atomic relaxation for large interchain separations, we build longer chains by connecting multiple chain blocks, performing numerical simulations for a discrete set of initial interchain (tail-to-tail) distances *h*_0_. For each block of size *L*, we solve the variational minimization problem, imposing that the difference between two consecutive initial interchain distances *h*_0_ is equal to twice the total vertical applied displacement at the right end of each chain (see Fig. 1c). This ensures overall geometric continuity, despite block discretization. To investigate the adhesive behavior at separations in the range [10–600 Å], the total length (with 40 blocks) of each wire is equal to 2.04 μm. The vdW interactions between different blocks are neglected.

The vertical displacement is applied by small loading increments to avoid large elongations of carbon bonds, and we assume that the wedge insertion is sufficiently slow, such that the geometry reaches an equilibrium state after each increment. It is possible that vertical displacements alone cannot fully model the effect of the wedge, due to co-existing horizontal and shear displacements between the layers (shear strain). We thus performed additional tests (see Supplementary Note 6 and Supplementary Fig. 3) to study the effect of the wedge, i.e., the sensitivity to the size of the applied vertical/horizontal displacement on the adhesive stress.

The TSL obtained by this numerical procedure is shown in Fig. 1. The effect of atomic relaxation on the adhesive stress is indeed substantially more noticeable than the previously considered effects. For instance, already at the PW vdW level, *σ*(*h*) exhibits slower decay and reaches negligible values only at 100 Å, i.e., extending the range of the interaction by a factor of three compared with adhesion of bodies with rigid atoms.

Remarkably, upon inclusion of MBD vdW interactions, the effect of geometry relaxation qualitatively differs from the approximate PW level: the adhesive stress range now extends beyond 500 Å, i.e. increasing by more than an order of magnitude with respect to the rigid geometry configuration. We will demonstrate below (see also Supplementary Fig. 2) that such an increase in the adhesive stress range stems from a delicate interplay between electronic and atomic degrees of freedom via wave-like atomic geometry deformations that persist up to large separations between materials. The results presented in the main part of Fig. 1 have been obtained with a conservative damping of the dipolar interactions within the MBD Hamiltonian (nearest-neighbor dipole interactions were excluded for the C chain, see details in Supplementary Note 1). Another test was performed to study the sensitivity to changes in MBD parameters by allowing natural oscillator-wavefunction damping for nearest-neighbor dipolar interactions, which remarkably lead to even longer adhesive stress range. The MBD area shown in the inset (a) of Fig. 1 illustrates the upper and lower bounds corresponding to these two sets of parameters, and clearly demonstrates the qualitative impact of MBD interactions for extending the distance range of the adhesive stress. The dependence of the adhesive stress with respect to distance in fully relaxed MBD geometries is in relatively good agreement with available experiments on graphene delamination from silicon^15^. Since MBD calculations and especially variational geometry relaxation of extended systems imply substantial computational cost, extrapolation of MBD TSL curves to infinite chain size and larger chain separations is currently unfeasible. Hence, comparison with experiments remains qualitative at this stage. However, it is readily observed from Fig. 1a that only MBD leads to sufficiently slow *σ*(*h*) decay, compared with ultra-long-ranged experimental curves. We will also show below that a 2D/3D MBD model would in fact further increase both the magnitude and the range of the adhesive stress, arguably leading to an even better agreement with experiments.

Variational calculations based on MBD vdW interactions yield best agreement with experiments and offer novel insights into ultra-long-range adhesive stresses in low-dimensional materials (see below). However, the main advantage of effective PW models for the vdW interaction over the explicitly quantum-mechanical MBD approach is their simple analytical expression, which implies high computational efficiency and applicability to much larger systems. Aiming to provide an effective PW potential that would reproduce the MBD results for 1D systems, we assessed different PW power laws and carried out for each power law full relaxations, effectively fitting the MBD TSL. The best fitted curve shown in Fig. 1 is obtained with the following potential:

1 $${E}_{{\rm{PW-fitted-to-MBD}}}=-\sum _{j> i}{\epsilon }_{ij}\frac{{\rm{0.79}}\cdot {\sigma }_{ij}^{4}}{{r}_{ij}^{4}}=-\sum _{j> i}\propto \frac{1}{{r}_{ij}^{4}},$$

where *r*_*ij*_ the distance between two atoms (in atomic units). The effective interatomic PW potential in the above equation now decays as ${r}_{ij}^{-4}$, substantially differing from the traditional ${r}_{ij}^{-6}$ LJ term, and effectively capturing the long-range quantum effects explicitly described by the MBD model. In fact, many-body effects are known to critically influence both intensity and power-law decay of the vdW interactions in low-dimensional nanostructures^27,31,41^, being consistent with the obtained parameterization. We also note that the effective PW power law and the associated coefficient will greatly vary depending on the dimensionality and geometry of the system under consideration; hence, the obtained ${r}_{ij}^{-4}$ vdW interaction power law is specific to the employed model of two 1D wires. Nevertheless, an extension of fitted effective pairwise interactions to different nanomaterials (to be addressed in future work) offers a promising direction to develop a systematic database of adhesive potentials for materials with different polarization properties, topologies, and dimensionalities.

## Analysis of physical parameters influencing the adhesive stress

In this section, we will provide a concise analysis of different physical parameters that influence the adhesive stress. This analysis will demonstrate that electronic charge-density fluctuations described by the MBD Hamiltonian interplay with atomic deformations, giving rise to wave-like geometrical patterns that are ultimately responsible for the observed long-range adhesive stress. Such deformations are absent or negligible in the localized pairwise vdW model, explaining the qualitative differences observed between PW and MBD approaches. In addition, aiming toward more realistic 2D/3D models, we performed MBD calculations for relaxed 2D geometry, with deformation modeled upon fully relaxed wire structures. The obtained results further reinforce our observations for wire models, i.e., the adhesive stress is enhanced both in magnitude and distance range for a 2D system. These additional tests demonstrate the robustness of our conclusions, and indicate that the observed long-range proximity effect is a general phenomenon for thin deformable membranes and flexible low-dimensional nanostructures, providing strong motivation for future adhesion experiments on systems of different dimensionality and topology.

**Fig. 2:**
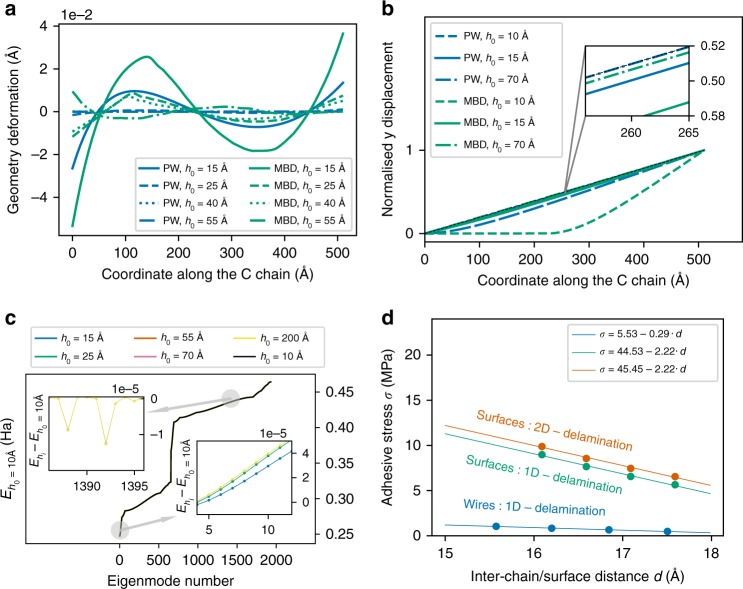
Analysis of physical parameters influencing the adhesive stress. **a** Secondary geometry deformations for different values of the interchain distance *h*_0_. Relaxed MBD geometries exhibit more pronounced deformations than those obtained from the PW model. MBD deformations slowly decrease at growing interchain distance, while the PW deformations quickly converge to zero for *h* ≥ 25 Å. **b** Carbon wires normalized *y* displacement in the relaxed geometry for different values of *h*_0_. Relaxed MBD geometries exhibit more significant non-linearities compared with PW. **c** Frequency spectrum of the collective electron-density oscillation modes, derived from the MBD Hamiltonian (plasmon dispersion) at various interchain distances *h*_0_. Frequency variation between a given interchain distance and *h*_0_ = 10 Å is reported for the lowest and highest energy mode intervals. **d** Adhesive stress as a function of the interchain/surface distance for three models with different dimensionality. Dimensional effects are clearly captured showing that the most complex 3D separation model (where both structures are allowed to delaminate in all directions) exhibits longer adhesive stress range and larger adhesive strength.

To quantitatively analyze the relaxed geometries obtained upon variational optimization, normalized C atom *y* displacements are shown in Fig. 2b for different values of the interchain distance *h*_0_. The analysis of this plot consistently reveals the existence of well-defined primary deformations, approximately corresponding to wave-like patterns with a wavelength corresponding to twice the length of one chain block (L = 510 Å). Primary deformations can also be viewed as the main, large-scale relaxed-chain deviation from the ideal linear geometry connecting the two-chain edges. Under equal loading conditions, relaxed MBD geometries exhibit substantially higher non-linearity (i.e., smaller curvature radius) compared with the corresponding PW-deformed structures. The observed non-linearity enhancement is to be attributed to the stronger and longer-ranged dispersion interactions^27,41^, arising in the quantum-mechanical MBD approach due to correlated many-body charge fluctuations. Increasing *h*_0_ gradually leads to weaker non-linearity, consistent with the decrease in interchain vdW interaction.

We further analyze our system by fitting optimized geometries via a quadratic polynomial, and determine secondary geometrical patterns as the difference between the actual relaxed geometries and smooth fitted polynomials (see Fig. 2a). Again, relaxed MBD geometries exhibit substantially larger secondary deformations with respect to PW. MBD-induced distortions slowly decrease at growing interchain distance, while PW deformations converge to zero already for *h*_0_ ≥ 25 Å. Such secondary deformations also exhibit wave-like patterns and correlate both with primary structure non-linearity and occurrence of ultra-long-ranged adhesive stress *σ*(*h*). Physically, formation of primary and secondary geometric distortions implies effective enhancement of the interchain vdW binding (see Supplementary Note 5), with consequent increase in the overall adhesive stress *σ*(*h*). However, while such deformations in principle exist both at the PW and MBD levels, mechanical deformations come at a non-negligible elastic energy cost, which can be efficiently released only when including many-body vdW interactions.

### MBD oscillation modes

Many-body vdW interaction enhancement occurring in low-dimensional nanostructures ultimately relates to the highly coherent motion of fluctuating electronic dipoles, and the consequent presence of slowly decaying oscillating electric fields. Hereafter, we report the energy eigenvalue spectra corresponding to the 3*N* collective eigenmodes of the many-body dispersion model, in ascending frequency order for two 1D wire models comprising 644 atoms, at interchain distances ranging from 10 to 200 Å (see Fig. 2c). These eigenmodes correspond to collective electronic polarizations with plasmonic character as discussed in detail in ref. ^27^. More precisely, the lower-energy modes are associated with electronic fluctuations extending over the whole chain, with the largest dipole oscillation components parallel to the chain axis. In contrast, higher-energy modes also involve hybrid fluctuations, which have finite components in directions parallel and perpendicular to the longitudinal axis.

Due to interchain dipole coupling, collective modes arising in the two chains interact, undergoing frequency shifts (and consequent total energy variations) that ultimately determine interchain vdW attraction. As from Fig. 2c, the frequency shifts due to interchain coupling (here computed by comparing different interchain distances) are small (of the order of 10^−5^ Ha). However, the vdW energy difference between distinct geometries involves integration over the whole frequency spectrum. Hence, in spite of the single modes undergoing modest shift, summation over about 2000 modes still leads to sizeable energy variation for different *h*_0_ values. We note that while in flat 1D chains the low-frequency longitudinal modes alone essentially determine the whole interfragment vdW interaction, in the present systems, the symmetry loss caused by primary and secondary patterns involves more complex dependence on mixed modes. In fact, structural curvature variations and interaction between nonparallel chain segments turn out to effectively enhance the relevance of transverse electronic oscillations. For instance, in Fig. 2c, we report frequency shifts between configurations with different *h*_0_, for selected spectral ranges both at high and low frequency. We observe that the lowest frequency modes are shifted upward in energy by an increase in the interchain distance, whereas an opposite, yet comparably relevant effect is found for the selected higher-energy eigenmodes. Physically, the coupling between distorted (nonparallel) chains tends to contrast the “coherence” of MBD longitudinal fluctuations occurring along each separate chain, increasing the frequency of the lowest eigenmodes. In contrast, transversal fluctuations can be stabilized (lowered in energy) by the interchain coupling, as they acquire a nonvanishing effective longitudinal component in curved geometries.

Overall, the highly collective wave-like character of both mechanical atomic deformations and many-body plasmon-like electronic oscillations unavoidably implies complex interplay between electrons and ions, which could not be captured within the widely used PW vdW approximation. In fact, the PW model has no underlying mechanism for delocalized response, either electronic or ionic. MBD-based coupled quantum mechanics/continuum mechanics therefore provides a major step forward, due to the naturally built-in cooperativity between charge fluctuations and geometrical deformations.

We also point out that long-range atomic deformations (lying in the infrared frequency region) can efficiently interact via the electromagnetic field with the quickly oscillating plasmonic modes^42,43^. In fact, while each of these collective charge oscillation modes lies in the ultraviolet-frequency region, the integral over plasmonic frequency shifts is situated in the infrared range. The coupling between quick many-electron oscillations and slow collective atomic vibrations is thus an essential ingredient of the present variational optimization, and ultimately determines the observed wave-like geometry patterns. Future work will concentrate on a systematic investigation of these cooperative coupling effects over a broader class of systems.

### MBD dimensional effects

To move beyond the 1D models studied with MBD up to this point, we now consider three different geometries, constructed taking a deformed 1D chain as shown in Fig. 2b as a starting point: (i) two wires modeled by only a one-dimensional linear delamination; (ii) a two-surface model with a directional delamination along the *z* axis described by *z*(*x*) = *ax* + *h*_0_; (iii) a two-surface model with delamination in both the *y* and the *z* axes described by a quadratic surface: *z*(*x*, *y*) = *ax* + *by*^2^ + *c*. For the surface models, we fixed all the atomic positions to calculate directly the vdW forces (without minimization to compute the optimal relaxed geometries). Both two-surface models offer approximate 2D/3D extensions of the relaxed two-chain model to the case of graphene delamination, thus providing comparison and validation of the above results in the context of membrane adhesion. More specifically, a cut of the surface by a plane perpendicular to the *y* axis (see Fig. 1e) gives a linear profile with the same slope as the linear fitting curve obtained from the results of the 1D-relaxed chains. For the profile *z*(*x*, *y*), the parameter *b* is fixed to study the effect of delamination along two different axes.

We investigated the adhesive stress as a function of the separation for a distance range between 15 and 18 Å, in order to probe how the slope and magnitude of the adhesive stress change from 1D to 2D models. For the two-surface models, 3936 carbon atoms and 774 silicon atoms are used. The 3D delamination model between the two surfaces is schematically illustrated in Fig. 1e. Carbon atoms are arranged following a hexagonal lattice with a lattice constant of 1.2 Å, while silicon atoms are arranged on a regular grid with a lattice constant of 2.35 Å. The adhesive stress is computed using the Cohesive Zone Volume Element (CZVE) described below and the results are presented in Fig. 2d. In our 1D calculations, the length of the CZVE is fixed as *l* = 60 Å. For the surface models, the CZVE is a surface of length *l* = 60 Å and width 25 Å.

The analysis of these preliminary calculations shown in Fig. 2d confirms the importance of dimensional effects and the fact that the full 2D/3D model increases both the strength and the range of the adhesive stress, offering stronger evidence for the agreement between our quantum/continuum model and experimental predictions.

## Discussion

We developed a coupled quantum/continuum model to calculate adhesive traction-separation laws for arbitrary nanomaterials via variational optimization, combining elastic theory of interatomic bonds with full inclusion of quantum-mechanical many-body vdW interactions. Application of the method to delamination of low-dimensional nanostructures—including 1D chains and 2D graphene—from Si substrates reveals a complex interplay between structural deformations and many-body vdW interactions, ultimately leading to ultra-long-ranged proximity effects. While the TSL produced by traditional pairwise vdW approaches underestimates the interaction range by three orders of magnitude compared with experiment, an appropriate account of quantum-mechanical many-body effects and the resulting geometry deformations substantially extends the distance range of the adhesive stress, providing qualitative agreement with experimental evidence. Physically, long-ranged quantum many-body vdW interactions result in an adhesion-enhancement mechanism, involving the emergence of coherent wave-like geometrical deformations. This effect implies cooperative interplay between atomic mechanical deformations and collective quantum-mechanical charge oscillations. Hence, widely adopted classical atomistic molecular dynamics simulations would entirely miss the emergent long-range proximity effect observed in this work.

Our work paves the way in the broad and largely unexplored domain of quantum/continuum simulations. Many points remain open for investigation to ensure that the proposed model actually reflects experimental conditions. For instance, our approach should be tested on higher-dimensional 3D nanostructures. Dimensional effects play a crucial role, as we qualitatively showed that full relaxation of a complex 3D model tends to better reconcile experimental observations with longer adhesive stress range and larger adhesive strength.

The role of defects (e.g., topological and vacancy defects) should also be investigated as these can alter the overall polarizability and influence, in a self-consistent manner, the global macroscopic behavior of the materials. Thermal effects were not considered in this work, but we speculate that finite temperature should in fact increase the adhesive stress in the presence of MBD vdW interactions. In fact, infrared vibrations of organic matter are amplified upon increasing the temperature^42,43^, and this suggests that electronic delocalization increases concomitantly. It would also be interesting to go to multilayer structures and study the mode mixity dependence of adhesive properties and fracture toughness^44^.

The quantum/continuum framework developed in this work can be used in a broad spectrum of applications, including the wear and tear of material interfaces, tribological properties of surfaces, mechanical properties of nanocomposites, or transport and behavior of biological membranes. This work makes a necessary step toward the construction of efficient multiscale methodologies, bridging directly quantum mechanics to continuum mechanics for describing stress and adhesion in complex nanoscale and mesoscale materials.

## Methods

### Methodology

We present here the methodology to extract the TSL. We model the experimental setup through two kinds of separation at the atomic scale: (a) simple approximation of the deformed geometry for interfacial separation, and (b) a variational method to characterize the deformed configuration where the geometrical complexity of both interfaces is taken into account. We also provide in Supplementary Note 2 a theoretical 3D approach to treat the flat separation between a surface (graphene) and a volume (silicon) with a pairwise potential.

### van der Waals dispersion interactions

Both pairwise (PW) and many-body (MBD) approaches are considered to compute the vdW forces at each particle. For the PW method, the vdW interactions are described by the attractive part of a Lennard–Jones (LJ) potential without damping function. In the second case, we adopt the MBD method^29^, a powerful approach for calculating the interatomic interaction energy based on the adiabatic connection fluctuation dissipation theorem (ACFDT) within the random-phase approximation (RPA) for a model system comprising quantum harmonic oscillators (QHO) interacting via the dipole–dipole interaction potential^45^. The MBD model generalizes the pairwise vdW energy expression by considering all orders of the dipole interaction between fluctuating atoms. Following the work of Ambrosetti et al.^30^, the ACFDT–RPA correlation energy for the MBD model is expressed as follows:

2 $${E}_{{\rm{c}},{\rm{MBD}}}=\frac{1}{2\pi }\int\nolimits_{0}^{\infty }{\rm{Tr}}\left[{\rm{ln}}({\bf{1}}-{\boldsymbol{AT}})\right]{\rm{d}}\omega ,$$

where ***A*** is a diagonal 3*N* × 3*N* matrix, which is defined as a function of the polarizability *α* and frequency *ω*. ${{\boldsymbol{A}}}_{lm}=-{\delta }_{lm}{\alpha }_{l}\left(i\omega \right)$ for the case of isotropic QHOs, and ***T*** is the dipole–dipole interaction tensor:

3 $${T}_{ij}^{ab}={\nabla }_{{r}_{i}}\otimes {\nabla }_{{r}_{j}}{v}_{ij}^{{\rm{gg}}},$$

*i*, *j* indicate atom *i* and atom *j*, *a* and *b* specify the Cartesian coordinates, and ${v}_{ij}^{{\rm{gg}}}$ is a modified Coulomb potential, used to incorporate overlap effects for a set of fluctuating-point dipoles

4 $${v}_{ij}^{{\rm{gg}}}=\frac{{\rm{erf}}({r}_{ij}/(\beta \cdot {\tilde{\sigma }}_{ij}))}{{r}_{ij}},$$

in which *r*_*ij*_ is the interatomic distance between atom *i* and *j*; ${\tilde{\sigma }}_{ij}$ an effective width, ${\tilde{\sigma }}_{ij}=\sqrt{{\sigma }_{ii}^{2}+{\sigma }_{jj}^{2}}$ computed from the atom’s Gaussian widths *σ*_*ii*_, *σ*_*jj*_ of atoms *i* and *j*, respectively. *β* is an empirical constant; a value greater than 1 corresponds to an interaction that is shifted to larger distances.

The interatomic forces are derived by taking the gradient of the energy defined in Eq. (2), which gives

5 $${{\boldsymbol{F}}}_{{\rm{c}},{\rm{MBD}}}=-\nabla {E}_{{\rm{c}},{\rm{MBD}}}=\frac{1}{2\pi }\int\nolimits_{0}^{\infty }{\rm{Tr}}\left[\right.\frac{\nabla {\boldsymbol{AT}}}{\left({\bf{1}}-{\boldsymbol{AT}}\right)}\left]\right.{\rm{d}}\omega .$$

The matrix ***A*** (and hence the polarizabilities) is assumed to be independent of the atomic positions so that the differentiation  ∇***AT*** can be efficiently performed via Gauss-Legendre quadrature^30^.

### Chemical binding energy

In the framework of unidimensional separation between two wires with small deformation, the bonding energy due to alteration of bond and torsion angles can be neglected. Hence, the bonding energy is assumed to be mainly due to the interaction between atomic pairs where atoms are separated by one covalent bond, which can be described by the pairwise harmonic potential

6 $${E}_{{\rm{b}}}=\frac{\gamma }{2}{\left(r-{r}_{0}\right)}^{2},$$

where *r*_0_ is the ideal equilibrium length at the initial state, *r* is the new equilibrium bonding length in the deformed state, and *γ* is the force constant, which determines the bonding strength.

### Flat separation between two wires

To model the interfacial delamination experiments carried out in ref. ^15^, we first build a simple 1D model involving two atomic wires, namely a carbon chain (426 C atoms) and silicon chain (218 Si atoms). The interfacial separation is here modeled by the interchain distance *h*. In this case, the geometry of each of the two atomic chains is constrained to remain flat, and we can directly compute the vdW forces for different values of *h*.

### Relaxed separation of two wires

Variational principle based on energy minimization is used to characterize the relaxed structure. The total energy *E* for the system containing the same two wires can be assumed as a summation of nonbonding energy *E*_nb_ and bonding energy *E*_b_:

7 $$E={E}_{{\rm{nb}}}+{E}_{{\rm{b}}}.$$

The nonbonding (vdW) energy *E*_nb_ is modeled either by PW or by MBD.

Boundary conditions are chosen as follows: for each chain block, the head atoms of the C/Si chain are gradually displaced in the *y* direction by repeated displacement increments $\Delta {u}_{y}^{{\rm{Si}}}=0.1$ Å and $\Delta {u}_{y}^{{\rm{C}}}=0.1$ Å, while the *x* displacements of these two atoms are fixed to zero due to symmetry. Tail atoms (left-hand side) are kept fixed along both *x-*  and *y* directions, at interchain distance *h*_0_ (see Fig. 1c). Knowing the full potential energy, we formulate a variational principle based on energy minimization to determine the deformed geometry of the atomic structure

8 $${\boldsymbol{x}}={\rm{Arg}}\left\{{\mathop {\mathrm{inf}}\limits_{\boldsymbol{x}}^{\,\!}}\left(E\right)\right\},$$

where ***x*** represents the atomic positions and *E* the total energy in the ***x*** configuration. The solution of this optimization problem yields optimized structures for PW or MBD interaction models after each displacement increment. To avoid unphysically large energy gradients within the atomic wires, applied displacement must be small compared with the structure size. The number of increments depends on the desired level of loading. A chain block has a length L = 510 Å. The whole chain model is composed of 40 such blocks, yielding a chain length of  2.04 μm. For each chain block, the chosen total applied displacement *u* corresponding to the insertion of a small wedge is equal to 7.5 Å (1.5% of the length of one chain block).

### Procedure to construct continuum traction-separation laws

The parameters used for the numerical simulations involved in the harmonic potential, the Lennard–Jones potential, and the MBD method are shown in Supplementary Table 1. We define the adhesive force ${F}_{y}^{i}$ on a given atom *i* in the carbon chain as the sum of all vdW forces exerted by all atoms in the system (both chains). This adhesive force is in equilibrium with the chemical binding forces. To compute the adhesive stress at atom *n* along the carbon chain, we sum the adhesive forces acting on all atoms *i* with 1 ≤ *i* ≤ *n*, where atom 1 sits at the clamped edge, and divide this resulting adhesive force by the total surface *nd*_C–C_*d*_0_:

9 $$\sigma =-\frac{1}{n}\mathop{\sum }\limits_{i=1}^{n}\frac{{F}_{y}^{i}}{{d}_{{\rm{C}}-{\rm{C}}}{d}_{0}},$$

where *d*_*C*–*C*_ is the distance between neighboring carbon atoms and *d*_0_ is a suitable length scale, fixed to 1 Å, which is a standard order of magnitude compared with the range of values commonly used^46^. To compute the adhesive stress, we do not take into account the atoms where the vdW force is above the threshold value of 0.001 nN. Hence, several atoms (typically three/four) located close to boundary atoms are removed . This prevents spurious oscillations due to boundary effects. First, the relaxed structure is used to compute vdW forces. Then, the cohesive zone volume proposed in ref. ^47^ is used as a basis to construct the traction-separation laws. The atomic structure is divided into a number of boxes containing several atoms, called the cohesive zone volume element (CZVE). We determine the cohesive parameters in each box. This approach leads to smooth cohesive properties along the chain, leading to a homogenized continuum traction-separation law. The CZVE concept has been successfully used in refs. ^48,49^ where the traction-separation law of interfaces has been used in other contexts. In our calculations, the length of the CZVE box is fixed as *l* = 60 Å. The corresponding crack opening is approximated as the average distance between two opposite CZVEs. We construct the TSL by applying Eq. (9) in the context of the CZVE technique. More details are provided in Supplementary Note 3.

## Supplementary information

Supplemental material

## Data Availability

All relevant data and code to reproduce the results presented in this paper are available from the corresponding author upon reasonable request.
