# Supplementary material for: From quantum to continuum mechanics in the delamination of atomically-thin layers from substrates
Source: Nat Commun. 2020 Apr 3;11:1651. doi: 10.1038/s41467-020-15480-w (PMC7125152; doi:10.1038/s41467-020-15480-w)
Supplement: Supplementary file 1 — Supplemental material [file 41467_2020_15480_MOESM1_ESM.pdf]

From Quantum to Continuum Mechanics in the Delamination of Atomically-Thin Layers from Substrates

Hauseux et al.

Supplementary materials

Supplementary Note 1 Numerical parameters

For the PW method, interatomic forces can be computed by taking the gradient of the LJ potential:

$$\boldsymbol{F}_{\mathrm{LJ,PW}} = -\nabla E_{\mathrm{LJ,PW}} = \sum_{j>i} 4\epsilon_{ij}\, \nabla \left(\frac{\sigma_{ij}^6}{r_{ij}^6} \right), \tag{1}$$

where r_{ij} indicates the interatomic distance between atom i and j; $\sigma_{ij} = 0.5 \cdot (\sigma_{ii} + \sigma_{jj})$ and $\epsilon_{ij} = \sqrt{\epsilon_{ii}\, \epsilon_{jj}}$ are numerical constants determined from the properties of atom i and j.

Parameters used for the numerical simulations involved in the harmonic potential, the Lennard-Jones potential and the MBD method for carbon and silicon atoms are shown in Supplementary Table 1. These parameters give the MBD relaxed curve in the main graph of Fig. 1 which also corresponds to the lower bound of the MBD area plotted in Fig. 1(a). All the analysis in this paper was performed with these parameters, using a L-BFGS-B method (*1*) to solve the minimization problem. This memory-efficient algorithm has the advantage to require only the gradient of the total energy, without involving computation of the "exact" Hessian matrix. To obtain the upper bound of this MBD area, analogous physical constants were employed in combination with a different solver (Newton-Raphson method), removing the short-range MBD interaction cutoff. In practice, the vdW interaction for each carbon atom was computed by taking into account the contribution of all the other atoms (no short-range cutoff), whereas in the previous case the contribution of the first neighbouring atoms (right/left) is removed.

	carbon	silicon
γ (Hartree $\cdot$ bohr^{-2})	0.65	1.2
r_0(Å)	1.2	2.35
C_6 (Hartree $\cdot$ bohr^6)	46.6	305
α (bohr^3)	12.0	37.0
$\mathrm{V}^{\mathrm{eff}}/\mathrm{V}^{\mathrm{free}}$	0.87	0.9
β(MBD)	1.0	1.0
σ_{ii} (bohr)	6.44	7.22
ϵ_{ii} (Hartree $\cdot$ 10^{-5})	12.27	43.44
$\epsilon_{ii,\mathrm{PW-fitted-to-MBD}}$ (Hartree $\cdot$ 10^{-5})	12.27	40.6
η_{C} (bohr^{-2})	0.053	-
η_{Si} (bohr^{-3})	-	0.015

Supplementary Table 1: Parameters used for the numerical simulations involved in the harmonic potential, the Lennard-Jones potential and the MBD method for carbon and silicon atoms.

Supplementary Note 2 Flat separation between surface and volume, theoretical approach

A three-dimensional model, containing one graphene layer (infinite surface) and an infinite silicon volume is considered. Therein, the discrete atomic structure of graphene and silicon are approximated by an equivalent homogeneous structure represented by density η_{C} and η_{Si}. Let h be the distance between these two layers.

Following (*2*), the adhesive interaction between graphene and silicon for the case of flat separation can be modelled by using PW based methods. The cohesive energy (per unit area), between the infinite surface of graphene and infinite silicon volume writes:

$$\phi_{\mathrm{PW}}(h) = 2\pi\eta_{\mathrm{C}}\eta_{\mathrm{Si}} \int_h^{\infty} V(r) r (r-h)\, \mathrm{d}r, \tag{2}$$

where r is the distance between an infinitesimal area of the graphene and a material point of the silicon substrate, while $V(r)$ is the potential energy due to vdW forces.

The adhesive stress is defined as the energy variation with respect to the crack opening v.

In the case where the Lennard-Jones 6-12 potential is adopted for $V(r)$, the adhesive stress is defined as:

$$\sigma_{\mathrm{PW}}^{\mathrm{Flat}} = 2\pi\eta_{\mathrm{C}}\eta_{\mathrm{Si}}\epsilon_{ij}\left[\frac{\sigma_{ij}^{6}}{(h+v)^{4}} - \frac{2\sigma_{ij}^{12}}{5(h+v)^{10}}\right], \tag{3}$$

where ϵ_{ij} is the bond energy at the equilibrium distance between atom i and j.

The TSL between the C-surface and the Si-Volume defined by Eq. (3) decays according to C/h^4. Note that there are strong dimensional effects for this kind of Traction-Separation Law. For the case of a C-surface/Si-surface, the cohesive stress becomes $\sigma_{\mathrm{PW}}^{\mathrm{Flat}} = 8\pi\eta_{\mathrm{C}}\eta_{\mathrm{Si}}\epsilon_{ij}\left[\frac{\sigma_{ij}^{5}}{(h+v)^{5}} - \frac{\sigma_{ij}^{11}}{(h+v)^{11}}\right]$, *i.e.* exhibiting a C/h^5 scaling.

Supplementary Note 3 Continuum traction-separation laws in the context of the CZVE technique

In the context of the Cohesive Zone Volume Element (CZVE) technique, the constant n (see Eq. 9 in the manuscript) represents the number of carbon CZVE boxes comprised between the crack tip and the position of the CZVE box where the crack opening is equal to h. In the same way, the term F_y in Eq. 9 (in the manuscript) refers to the average vdW force in direction y calculated from all the vdW forces of the atoms located inside the CZVE. A portion of the TSL for crack opening around 60 Å is presented (for PW and MBD) in Supplementary Fig. 1 to show the influence of the CZVE technique when we calculate the adhesive stress. The CZVE approach leads to smooth cohesive properties along the chain by providing an average continuum TSL. This is particularly relevant for MBD calculations, where fluctuations in the force distribution are more pronounced with respect to the PW case.

In order to investigate the adhesive behavior at separations in the range $[10\text{ Å} - 600\text{ Å}]$ we perform numerical simulations for a discrete set of initial interchain (tail-to-tail) distances h_0. More precisely, for each block of size L we solve the variational minimization problem,

imposing that the difference between two consecutive initial interchain distances h_0 is equal to $2u$ where u is the total applied displacement at the right end of each chain (see Fig. 1(c)). This ensures overall geometric continuity, despite block discretization. The interaction between different blocks is neglected, given the large block size ($L = 510$ Å), and the first interchain distance is fixed to 10 Å, i.e. close to the known equilibrium interwire separation. Since we neglect the interaction between neighboring atoms, we only take attractive vdW force components into account (no damping function is required for both PW and MBD). Considering that the two chains unavoidably attract each other, we constrain our solver to always keep the interchain distance larger than 10 Å. We compute the TSL from the position (crack tip) whereabouts the interchain distance becomes larger than 10 Å. Contributions from first neighbouring atoms at right/left edges are also neglected in the minimization problem for the carbon chain. Moreover, when addressing the traction separation law, we compute the vdW force acting on each carbon by only taking into account the contributions coming from all other atoms (carbon and silicon) located at a distance larger than $7\ \mathrm{bohr}$ (short-range cutoff).

Boundary effects are clearly observed for the adhesive force distribution. In fact, there is a gap between the adhesive forces of the boundary and central atoms. The force gap within the MBD framework is smaller than in the PW approach. Moreover, the evolution of the adhesive forces from edge to central atoms is much smoother with the MBD method compared to the PW case. This means that the PW method has higher sensitivity to boundary conditions. We assume that this behavior is due to the complexity of the relaxed MBD geometry which allows the propagation of boundary forces to the central atoms and therefore makes it possible to capture long-range interactions.

We first use the CVZE technique to compute the average stress in each CZVE box. Then we average over the different boxes (from left to right). The total chain is divided into different blocks (one block is one chain of length L) and each block is divided into different CZVE

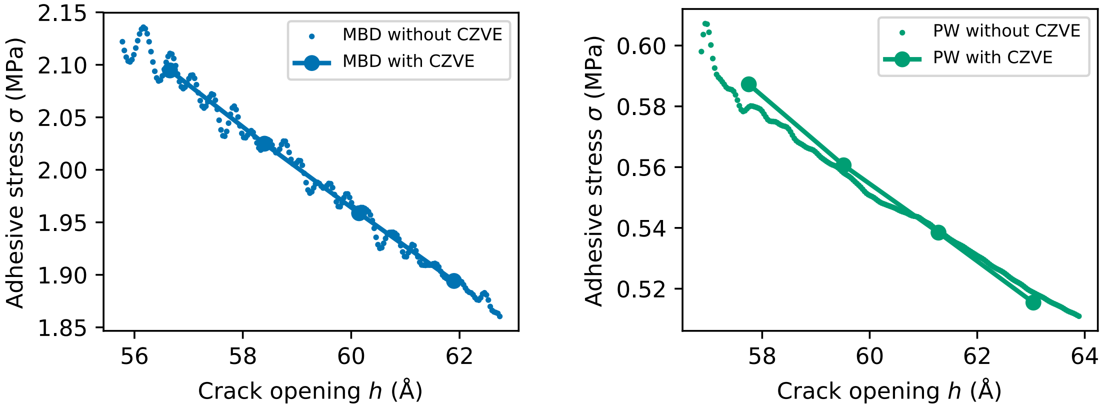

Supplementary Figure 1: Comparison of numerical predictions with and without the Cohesive Zone Volume Element (CZVE) technique for calculation of the adhesive stress. Only a portion of the TSL for crack opening around 60 Å is presented (for PW and MBD). The CZVE approach leads to smooth cohesive properties along the chain (for both PW and MBD) by providing an average continuum TSL along a characteristic length of the interface. Oscillations and fluctuations in the force distribution with the MBD method are more pronounced compared to PW.

boxes (fragments). The stress value for a given interchain distance h will be close but slightly different than the value obtained from a simple average of local/force area along the chain. The difference will depend on the length of the CVZE box. The advantage of our methodology is that we provide a smooth TSL curve without local fluctuations (see Supplementary Fig. 1).

Quantum-mechanical (QM) interactions in general and QM stress in particular are inherently non-local effects. QM stress behavior is ultimately determined by long-ranged fluctuations of electrons and ions and their mutual coupling. While “local projection” of the non-local quantum-mechanical stress is conceptually useful, it necessarily implies some “classicalization” procedure. Moreover, “local projection” techniques are highly non-unique: in fact, several averaging approaches can be defined, for instance by adopting different CZVE choices.

Notably, within the model adopted in our manuscript, one can prove that the average over chain-block vertical stress contributions equals the derivative of the average energy per block

with respect to h (i.e. the TSL defined in (*3*)) in the limit of long chains. This equality is easily demonstrated recalling that crack opening enlargement by $2u$ implies the introduction of an additional chain block. In our case, a very large (2 micrometer long) structure was taken into consideration for TSL computation at large crack openings h.

We also remark the TSL curves reported in (*3*) rely on indirect estimates of the crack opening, inferred by interference between light reflected from top and bottom crack surfaces. As reported by the authors, the experimental setup allows for resolutions of about 20 nm, which unavoidably limit the accuracy of reported TSL curves. For these reasons, a comparison between theoretical and experimental data can only be qualitative at this stage, which is clearly stated in our manuscript.

To address QM non-locality in more detail: dispersion interactions have non-local character and depend on charge fluctuations that essentially span the whole structure. This can be directly understood from the adiabatic connection fluctuation-dissipation formula within the random phase approximation (equivalent to MBD in the long-range limit). In fact, by taking a second-order perturbative approximation with respect to the interchain Coulomb coupling v, and writing the "dressed" density-density response functions of chains 1 and 2 as $\chi^1_{1,2}$, one can write the vdW interaction energy as:

$$\mathrm{E}_{\mathrm{c,vdW}} = -\frac{1}{2\pi}\int_0^\infty d\omega \int_{\mathrm{chain1}} d\mathbf{r}_1 d\mathbf{r}'_1 \int_{\mathrm{chain2}} d\mathbf{r}_2 d\mathbf{r}'_2 [\chi^1_1(\mathbf{r}_1, \mathbf{r}'_1, i\omega) v(\mathbf{r}'_1, \mathbf{r}'_2) \chi^1_2(\mathbf{r}'_2, \mathbf{r}_2, i\omega) v(\mathbf{r}_2, \mathbf{r}_1)], \tag{4}$$

where coordinate vectors $\mathbf{r}_{1,2}$ and $\mathbf{r}'_{1,2}$ run over chains (1,2), respectively. Due to the long-rangedness of $\chi^1_{1,2}$, the whole structure significantly contributes to the integral, making the whole vdW interaction energy extremely non-local.

As discussed throughout the paper, geometrical deformations also play a major role in determining the overall interchain adhesive stress. In this context, the concept of "local stress" has limited validity, as its details will essentially depend on hardly controllable non-local quantities.

One expects that small variations in the experimental conditions could lead to major changes in "local stress" properties, compatibly with (*3*).

We also remark that continuum elasticity theory is unable to predict the crack tip behavior, which makes the concept of "local" stress at the angstrom scale even more questionable. One usually introduces a cohesive zone model with experimentally calibrated parameters to link the macroscopic and atomistic scales. The main advantage of this approach is its direct applicability within a continuum mechanics framework, neglecting detailed physical effects occurring at the atomistic scale around the crack tip. In the present paper, instead, we directly modeled the crack tip, providing the TSL as a direct outcome of molecular dynamics simulations.

In addition, we underline that, within a coarse-grained approximation, the adopted average over computed piecewise stress contributions tends to the full energy derivative with respect to the rightmost crack opening h (i.e. the TSL as defined by (*3*)), in the limit of large cracks. In fact, within our model a crack vertical enlargement by $\delta h = 2u$ implies the addition of a chain fragment (crack height and length increase simultaneously). If the total energy E is the sum of piecewise terms $E = \sum_{i=1}^{N} E_i$, then variation of crack height (h) by δh leads to $E' = \sum_{i=1}^{N+1} E_i$. Then $dE/dh = \sum_{i=1}^{N} dE_i/dh + E_1/\delta h$. The average stress per fragment is thus approximately $(\sum_{i=1}^{N} dE_i/dh + E_1/\delta h)/N$, and this expression tends to $(\sum_{i=1}^{N} dE_i/dh)/N$ for large N (large crack limit). Hence, within the adopted approximations and at large h, the stress values plotted in Fig. 1 become equivalent to the TSL definition given in (*3*).

Supplementary Note 4 Direct evaluation of the vdW energy from the MBD model Hamiltonian

According to Ref. (*4*) the ACFD-RPA correlation energy defined by Eq. 5 (in the manuscript) can be directly obtained (for frequency independent $\boldsymbol{T}$ tensor) by diagonalization of a model Hamiltonian based on atom-centered QHOs coupled by dipole-dipole interaction. The inter-

action energy can thus be directly evaluated from the $3N$ (for N atoms) eigenvalues λ_i of the matrix composed by N^2 3×3 blocks $\boldsymbol{C}_{ij}^{\mathrm{MBD}}$ which characterize the coupling between each pair of atoms i and j:

$$\boldsymbol{C}_{ij}^{\mathrm{MBD}} = \omega_i^2 \delta_{ij} + (1 - \delta_{ij})\, \omega_i \omega_j \sqrt{\alpha_i \alpha_j}\, \boldsymbol{T}_{ij}. \tag{5}$$

In Eq. 5, α_i and ω_i are the isotropic dipole polarizability and characteristic frequency for atom i. These constants are determined as follows (*5*):

$$\alpha_i = \alpha_i^0 \cdot \frac{V_i^{\mathrm{eff}}}{V_i^{\mathrm{free}}};\ \omega_i = \frac{4\, C_{6,i}}{3\, (\alpha_i^0)^2}, \tag{6}$$

where α_i^0 and $C_{6,i}$ are the free-atom polarizability and C_6 terms. The factor $V_i^{\mathrm{eff}}/V_i^{\mathrm{free}}$ represents the ratio of the effective volume occupied by the atom interacting with its environment to the free (non-interacting) reference volume occupied by this atom. The MBD energy is finally computed as the difference between the interacting and non interacting frequencies:

$$E_{\mathrm{c,MBD}} = \frac{1}{2} \sum_{i=1}^{3N} \sqrt{\lambda_i} - \frac{3}{2} \sum_{j=1}^{N} \omega_j. \tag{7}$$

Supplementary Note 5 MBD analysis of wavelike geometry configurations

To better rationalize the emergence of non-linear primary and secondary patterns in MBD optimized chain configurations, we analyze in more detail their energetic impact. In order to simplify the problem, we report in Supplementary Fig. 2 the relative binding energy variation for parallel 1D carbon chains, upon introduction of regular wavelike distortion patterns. Intercarbon distance and Hirshfeld volume ratio are fixed according to Supplementary Table.1. As expected, we observe that the introduction of counter-phase half-wavelength primary patterns in the two chains causes sizeable vdW binding enhancement, due to increased vicinity between central chain areas. The effect gradually diminishes at large h_0, where the oscillation amplitude

scale becomes negligible with respect to the interchain distance. A qualitatively similar picture is found also at the PW level. However, due to the slower decay of many-body vdW interactions with respect to distance, we find that the MBD interchain binding energy at $h_0 = 40$ Å is 43 % larger than the corresponding PW value. Moreover, even stronger and longer-ranged interchain binding (more than an order of magnitude larger at h_0=40 Å) is found at the MBD level by including nearest-neighbor interactions, as a consequence of stronger many-body effects. Despite coexistence of both closer and farther chain segments, shorter-wavelength deformations still imply non-negligible binding enhancement with respect to the flat conformation, while minor binding variation is found between one- and two-wavelength oscillating patterns. In contrast, the overall binding reduction from half-wavelength to shorter-wavelength patterns justifies the observed slow oscillation observed in the primary structure. Hence, smaller, yet non-negligible secondary deformations can be viewed as a second-order effect, which is superimposed to the principal structure.

Interestingly, although the curvature relative to both primary and secondary structures may in principle contrast the coherence of many-body charge oscillation due to dipole misalignment, such effect remains negligible with respect to the many-body binding enhancement. The underlying reason is that the effective wavelengths characterizing both primary and secondary patterns are at least two orders of magnitude larger than the typical interatomic distance, and the oscillation amplitude scale is smaller than the chain length. This observation is independently confirmed by the above energetic analysis, and by the overall frequency spectrum structure, which is preserved regardless of the geometry relaxation occurring upon h_0 variation.

We finally recall that the conservative neglect of nearest-neighbor dipole coupling in MBD implies weaker mode coherence and diminished many-body vdW enhancement. For instance, lowest-frequency modes are lowered in energy by almost an order of magnitude in the presence of nearest-neighbor coupling, which is ultimately reflected in the (longer-ranged) MBD upper

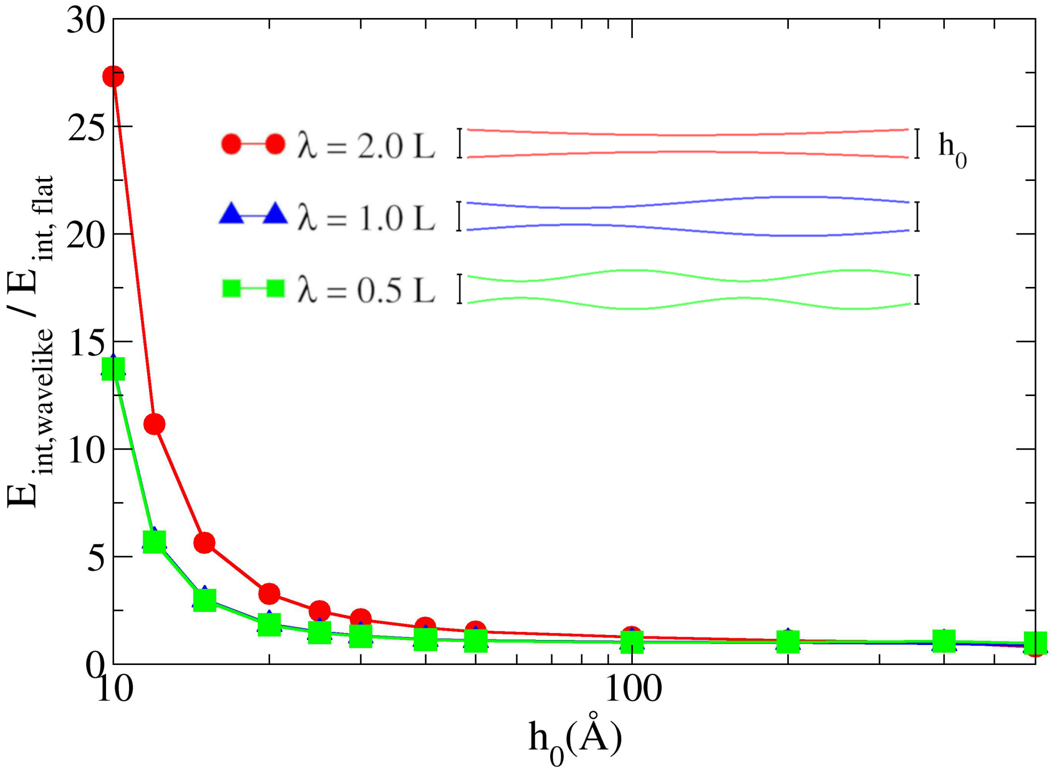

Supplementary Figure 2: Ratio between MBD interchain vdW binding in curved and flat geometry. Wavelike deformation patterns with different wavelengths λ (given as a function of the chain size L) are introduced. A supercell containing 200 C atoms per chain is adopted, in combination with periodic boundary conditions. Chain ends, indicated with black segments, are fixed at relative distance h_0 (given in logarithmic scale between 10 and 600 Å). Deformation amplitudes are set to 2 Å, in qualitative agreement with observed secondary structures. Higher energy enhancement is obtained at larger wave amplitudes.

bound for the adherence stress reported in Fig. 1 (a). The neglect of nearest-neighbor coupling is presumably exceedingly conservative, so that the actual adhesive stress range curve is eventually expected to lie closer to the upper bound. We observe, however, that even with underestimated many-body effects, MBD still displays major quantitative and qualitative difference from PW. Long-range proximity effect hence emerges as a robust physical mechanism, whose occurrence is ultimately independent from model parametrization.

Supplementary Note 6 Effect of the wedge, sensitivity to the size of the applied vertical/horizontal displacement.

We discuss in this part the influence of the applied vertical/horizontal displacement on the adhesive stress. As mentioned below, we chose to model the insertion of a thin wedge by only applying vertical displacements (1.5% of the length of the chain, $d = 7.5$ Å) to the right-hand side edge atoms. However, it is possible that small vertical displacements alone cannot fully model the effect of the wedge: in fact, horizontal deformations and horizontal relative displacements between the two layers (shear) may also be encountered. In addition, it is expedient to study the effect of applied vertical displacements (boundary conditions) over a reasonable variability range, given that the actual experimental deformations are ultimately unknown.

To model the horizontal wires displacement caused by wedge insertion, one should apply a slight longitudinal compression to the chains. This procedure, however, makes the variational geometry optimization extremely challenging, due to obviously increased complexity of the energy landscape. For this reason, we followed the opposite strategy, applying instead a finite tension to the chains (elongation of 5% for each wire, value taken as illustrative only). The trends obtained upon elongation are the expected to be roughly opposite to those caused by horizontal compression. A single compression test (at small vertical displacement) is also performed for confirmation. For a single block (2 wires) with initial interchain distance equal

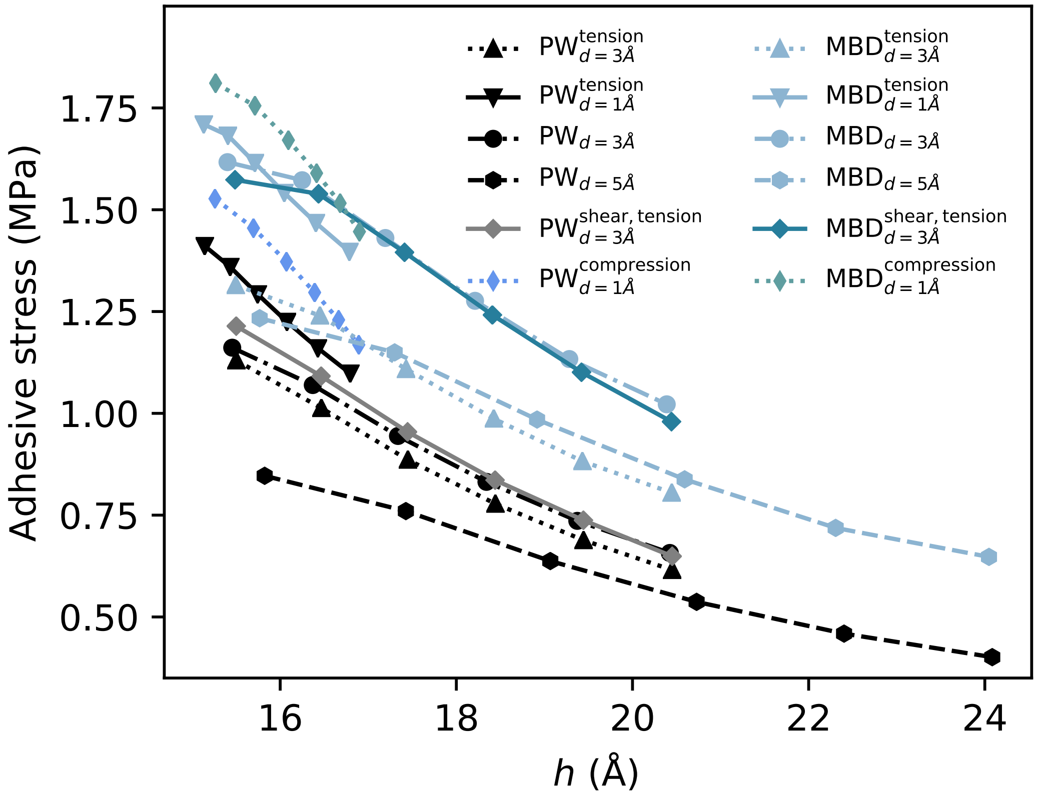

Supplementary Figure 3: Effect of the wedge: sensitivity to the size of the applied vertical/horizontal displacement. We apply an external tension (5% elongation for each wire - the value is taken as illustrative only) to model a horizontal displacement of the wires due to the insertion of a thin wedge during the delamination process. We also study the case of an external compression (5%). We compare the value of the adhesive stress at different vertical displacements d with and without initial tension or compression. Shear strain (relative horizontal displacement between carbon and silicon) is also considered. We notice that the boundary conditions can influence the adhesive stress. As expected, the adhesive stress decreases at larger applied vertical displacements d (size of the wedge) and tends to decrease when an external tension is imposed on the wires. Wire compression due to wedge insertion is thus expected to cause a net stress enhancement. Our numerical test confirms this tendency but we made only a single test under compression (for $d = 1$ Å) due to convergence difficulties. The higher sensitivity of MBD with respect to PW to horizontal stress is attributed to the decrease of many-body effects caused by larger interatomic separations. The MBD adhesive stress is always larger compared to the corresponding PW value regardless of the boundary conditions used to simulate the insertion of the wedge.

to 15 Å, we evaluate (see Supplementary Fig. 3) the adhesive stress at different applied vertical displacements d, with and without initial tension. We also study the case of shear strain when only the carbon wire is subject to horizontal displacement. Note that the TSL presented in the main part of the paper is calculated with an initial interchain distance equal to 10 angstroms. We kept the same loading increment to solve the minimization problem (0.1 Å per iteration). Finally, to obtain these curves, we facilitated numerical convergence by removing the short-range MBD interaction cutoff (as done in computing the upper bound of the MBD area shown in Fig. 1), and imposing a slightly higher parameter $\beta = 1.05$ in order to compensate for this choice. We remark that the qualitative conclusions of this work are independent of the approximations employed for the description of local elastic interactions. Results are instead somewhat sensitive to the adopted vdW damping parameters as shown in Fig. 1, but these do not alter the overall conclusions of our work. From Supplementary Fig. 3 we observe an effect of the type of boundary conditions used to simulate the insertion of the wedge on the adhesive stress.

As expected, we find that the adhesive stress tends to decrease with the applied vertical displacement (size of the wedge), due to overall increased interwire distance. We recall, however, that the choice of applying a constant vertical displacement to each chain segment represents a prudential approximation. Moreover, stress decrease is also found upon wires elongation. Hence, by contrast one reasonably expects net stress increase upon wedge-induced compression (as confirmed by the explicit compression test at $d = 1$ Å). Our numerical test confirms this tendency but we made only a single test under compression (with small vertical displacement $d = 1$ Å) due to convergence difficulties. We also observe that the PW method is slightly less sensitive to the applied horizontal displacement in comparison with the MBD approach, which is attributed to diminished many-body correlation effects caused by larger intrachain interatomic distances. The sensitivity to the horizontal displacement and to shear strain are similar between the two methods. Most importantly, regardless of the boundary conditions used to

simulate wedge insertion, the MBD adhesive stress is always larger than the corresponding PW value at any given interchain distance.

Supplementary Note 7 Analysis of the flat separation model

Although moderately larger stress range is found in the flat chain model upon inclusion of many-body effects with respect to the PW approach, MBD and PW methods are radically different. While PW approaches rely on a local description of the electron density response, MBD predicts highly collective dipolar response in low-dimensional nanostructures. This implies slower power law decay of the MBD interaction, and different reaction to geometrical deformations. In Supplementary Fig. 4, we report the adhesive stress at different interchain distances h for a structure of size $L = 510$ Å containing two flat and parallel chains: a carbon chain (426 C atoms) and a silicon chain (218 Si atoms). The inclusion of quantum MBD interactions leads to stronger adhesive stress at small interchain separations, in particular when including nearest-neighbor dipolar interactions by adoption of the natural oscillator-wavefunction damping. Within the PW flat model, instead, the value of the vertical force (y-component) does not vary upon inclusion of intrachain nearest-neighbor interactions. For large interchain separations, the adhesive stress is very small and almost negligible for both MBD and PW. We plot in Supplementary Fig. 5 the adhesive stress ratio between MBD and PW models to compare the different methods for large values of h. The inclusion of quantum MBD interactions for large interchain separations leads to stronger but very small in magnitude adhesive stress. So as mentioned above, we can say that only a slightly larger stress range is found in the flat chain model upon the inclusion of many-body effects.

In Supplementary Fig. 6, we plot the total force (projected onto the y-direction) acting on each carbon atom along the chain for $h = 10$ Å. Boundary effects are observed on the right and left sides, but the force is globally almost constant along the wire. As mentioned above, the vdW

forces obtained with the MBD model are larger than those predicted by the PW approximation.

As shown in (*6*), the power laws which govern vdW interactions between two parallel chains predicted by standard PW approaches have to be strongly modified when quantum MBD interactions are included in the model. In fact, although interaction ranges are apparently similar, the relative difference between the MBD and PW adhesive stress steadily increases with respect to h. Moreover, the inner mechanisms that regulate interatomic forces and their dependence on geometrical parameters in the PW and MBD approaches are profoundly dissimilar. To support our statement, we consider a carbon atom situated at x = 240 Å, and analyze in detail all the vertical force contributions that act on this atom. According to the PW model, one has N contributions, where N is the total number of atoms, as plotted in Supplementary Fig. 7. Due to the intrinsic locality of the PW density response, only a limited number of atoms in the structure provide a significant energy contribution. It is important to remark that all these contributions have negative (attractive) sign. On the other hand, within the MBD approach, one has $(3N)$ contributions corresponding to the collective modes of the system. As from Supplementary Fig. 7, these contributions can have either positive or negative sign, and have strongly non-local character. The balance between positive and negative terms ultimately determines the overall force and shows non-trivial dependence on the actual geometrical configuration. As a result, atomic displacements can a have strongly non-local impact on interfragment dispersion interactions, and major interplay arises between geometrical deformations and plasma-like charge fluctuations.

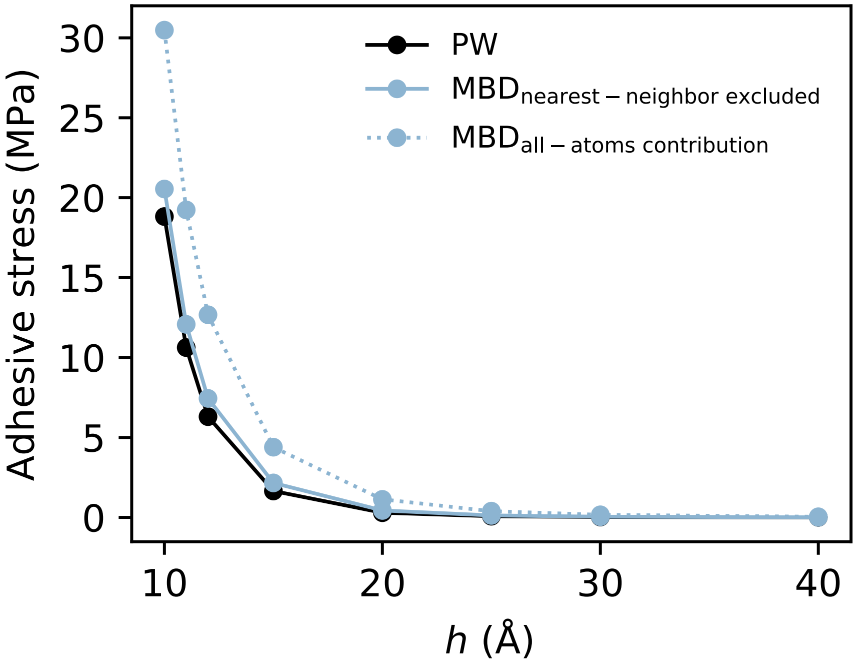

Supplementary Figure 4: Flat model: adhesive stress for different interchain distances h for a structure of size $L = 510$ Å containing two flat and parallel chains: a carbon chain (426 C atoms) and a silicon chain (218 Si atoms). Comparison between PW and MBD models. The results presented for the MBD model are obtained with two different dampings of the dipolar interaction. In the first case, we exclude nearest-neighbor dipole interactions within the C chain, while in the second case we adopt the natural oscillator-wavefunction damping for nearest-neighbor dipolar interactions. The inclusion of quantum MBD interactions in a flat-separation model leads to stronger adhesive stress, especially upon inclusion of nearest-neighbor interactions. Although the interaction range is apparently analogous in the different methods, the MBD stress follows a slower power law decay.

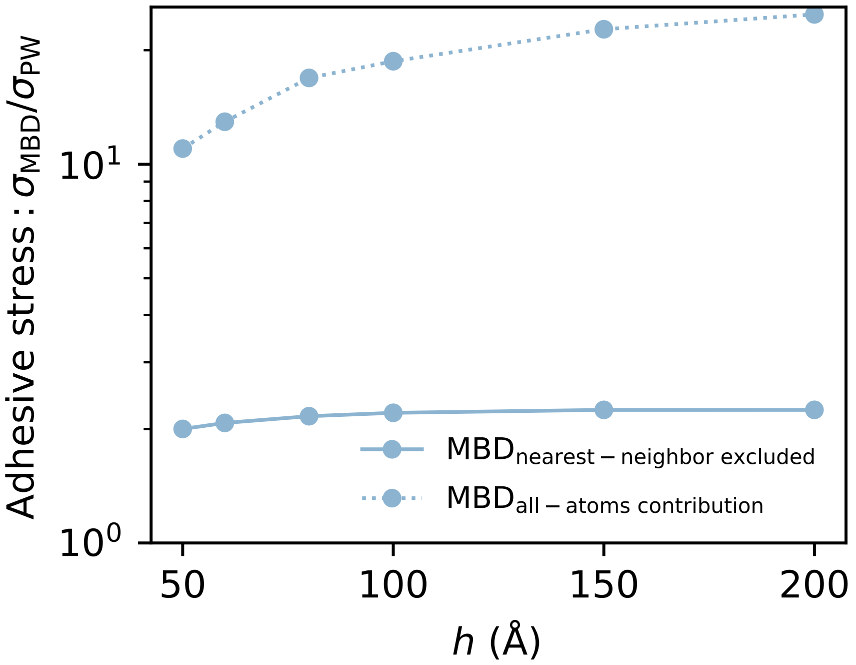

Supplementary Figure 5: Flat model: adhesive stress for different large interchain distances h for a structure of size $L = 510$ Å containing two flat and parallel chains: a carbon chain (426 C atoms) and a silicon chain (218 Si atoms). For large interchain distances, the adhesive stress is very small so that we plot the adhesive stress ratio between MBD and PW models to compare the different methods. The results presented for the MBD model are obtained with two different dampings of the dipolar interaction. In the first case, we exclude nearest-neighbor dipole interactions within the C chain, while in the second case we adopt the natural oscillator-wavefunction damping for nearest-neighbor dipolar interactions. The inclusion of quantum MBD interactions for large values of h leads to stronger but very small in magnitude adhesive stress.

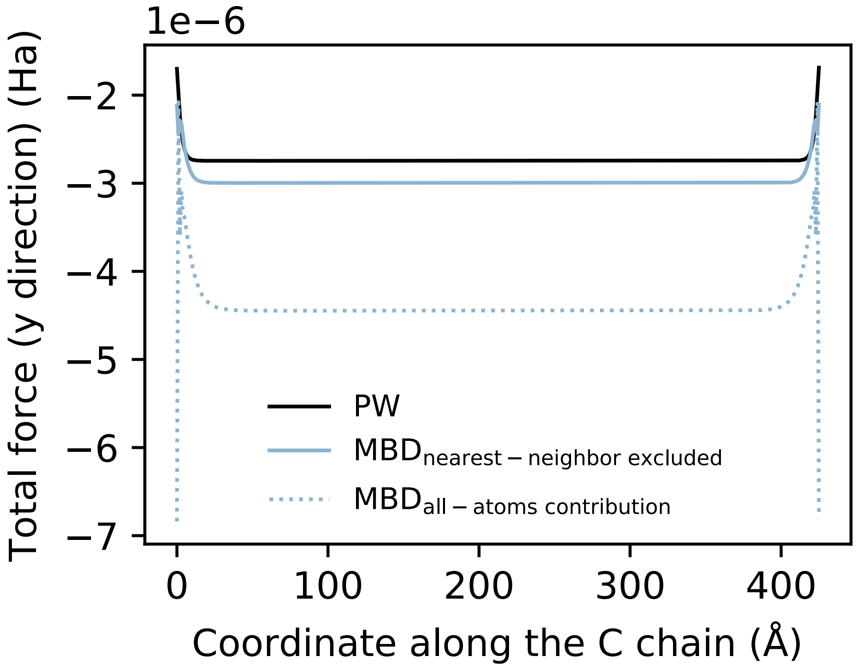

Supplementary Figure 6: Flat model for interchain distance $h = 10$ Å. Comparison between PW and MBD approaches for the total vertical force (y-component) which acts on each carbon atom along the chain. On the right and left sides boundary effects are present, however, the force is globally constant along the internal part of the wire. The inclusion of quantum MBD interactions in a flat-separation model leads to stronger adhesive stress (larger vertical forces on each C atom). The MBD adhesive stress is even larger when nearest-neighbor interactions are included by adoption of the natural oscillator-wavefunction damping.

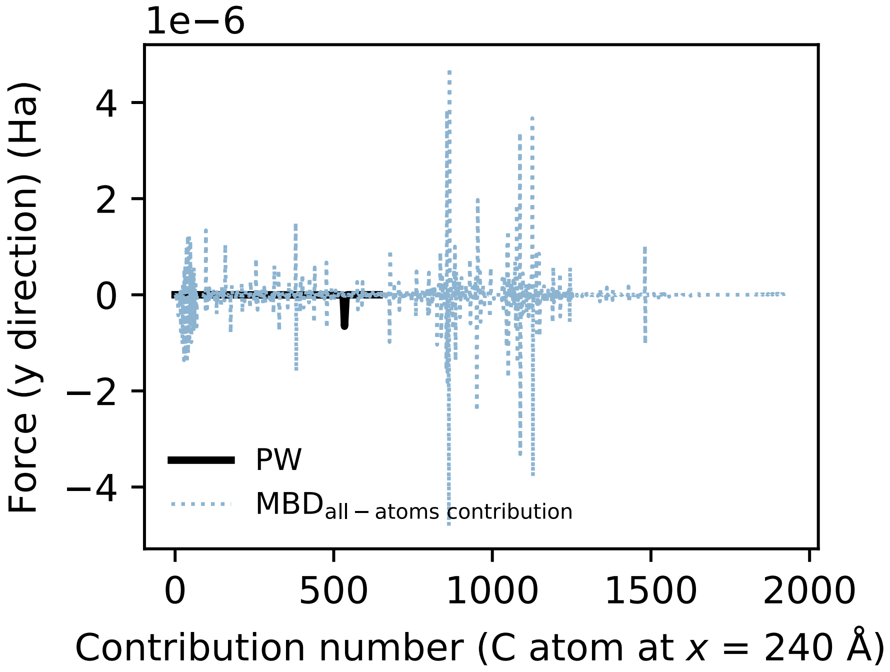

Supplementary Figure 7: Detailed vertical force contributions (y-component) acting on a carbon atom situated at x = 240 Å for interchain distance $h = 10$ Å. There are in total N atomic contributions for the PW model and $3N$ modes contributing to the MBD force. Due to the locality of the PW method, only a few atoms in the structure significantly contribute to the vertical force. All PW contributions have the same sign (negative). On the other hand, both negative and positive contributions arise in the MBD approach. The balance between positive and negative terms determines the overall force, and depends in a non-local fashion on the detailed geometry of the system.

Supplementary References

1. C. Zhu, R. H. Byrd, P. Lu, J. Nocedal, Algorithm 778: L-bfgs-b: Fortran subroutines for large-scale bound-constrained optimization, *ACM Trans. Math. Softw. (TOMS)* **23**, 550–560 (1997).

2. L. Y. Jiang, *et al.*, A cohesive law for carbon nanotube/polymer interfaces based on the van der Waals force, *J. Mech. Phys. Solids* **54**, 2436–2452 (2006).

3. S. R. Na, J. W. Suk, R. S. Ruoff, R. Huang, K. Liechti, Ultra long-range interactions between large area graphene and silicon, *ACS Nano* **8**, 11234–11242 (2014).

4. A. Ambrosetti, A. M. Reilly, R. A. DiStasio Jr., A. Tkatchenko, Long-range correlation energy calculated from coupled atomic response functions, *J. Chem. Phys.* **140**, 18A508 (2014).

5. A. Tkatchenko, M. Scheffler, Accurate molecular van der Waals interactions from ground-state electron density and free-atom reference data, *Phys. Rev. Lett.* **102**, 073005 (2009).

6. A. Ambrosetti, N. Ferri, R. A. DiStasio Jr., A. Tkatchenko, Wavelike charge density fluctuations and van der Waals interactions at the nanoscale, *Science* **351**, 1171–1176 (2016).
